# Community participation in formulating the post-2015 health and development goal agenda: reflections of a multi-country research collaboration

**DOI:** 10.1186/s12939-014-0066-6

**Published:** 2014-10-10

**Authors:** Claire E Brolan, Sameera Hussain, Eric A Friedman, Ana Lorena Ruano, Moses Mulumba, Itai Rusike, Claudia Beiersmann, Peter S Hill

**Affiliations:** School of Population Health, Faculty of Medicine and Biomedical Sciences, The University of Queensland, Brisbane, Australia; James P Grant School of Public Health, BRAC University, Dhaka, Bangladesh; O’Neill Institute for National and Global Health Law, Georgetown University, Washington, DC USA; Center for International Health, Department of Global Public Health and Primary Care, Faculty of Medicine and Dentistry, University of Bergen, Bergen, Norway; Centro de Estudios para la Equidad y Gobernanza en los Sistemas de Salud (CEGSS), Guatemala City, Guatemala; Center for Health, Human Rights and Development (CEHURD), Kampala, Uganda; Community Working Group on Health (CWGH), Harare, Zimbabwe; Institute of Public Health, Ruprecht-Karls University Heidelberg, Heidelberg, Germany

**Keywords:** Post-2015 agenda, Millennium development goals, Community engagement, Qualitative research, Reflexive analysis

## Abstract

Global discussion on the post-2015 development goals, to replace the Millennium Development Goals when they expire on 31 December 2015, is well underway. While the Millennium Development Goals focused on redressing extreme poverty and its antecedents for people living in developing countries, the post-2015 agenda seeks to redress inequity worldwide, regardless of a country’s development status. Furthermore, to rectify the UN’s top-down approach toward the Millennium Development Goals’ formulation, widespread negotiations are underway that seek to include the voices of people and communities from around the globe to ground each post-2015 development goal. This reflexive commentary, therefore, reports on the early methodological challenges the Go4Health research project experienced in its engagement with communities in nine countries in 2013. Led by four research hubs in Uganda, Bangladesh, Australia and Guatemala, the purpose of this engagement has been to ascertain a ‘snapshot’ of the health needs and priorities of socially excluded populations particularly from the Global South. This is to inform Go4Health’s advice to the European Commission on the post-2015 global goals for health and new governance frameworks. Five methodological challenges were subsequently identified from reflecting on the multidisciplinary, multiregional team’s research practices so far: meanings and parameters around qualitative participatory research; representation of marginalization; generalizability of research findings; ethical research in project time frames; and issues related to informed consent. Strategies to overcome these methodological hurdles are also examined. The findings from the consultations represent the extraordinary diversity of marginal human experience requiring contextual analysis for universal framing of the post-2015 agenda. Unsurprisingly, methodological challenges will, and did, arise. We conclude by advocating for a discourse to emerge not only critically examining *how* and *whose* voices are being obtained at the community-level to inform the post-2015 health and development goal agenda, but also how these voices are being translated and integrated into post-2015 decision-making at national and global levels.

## Introduction

**Figure 1:**
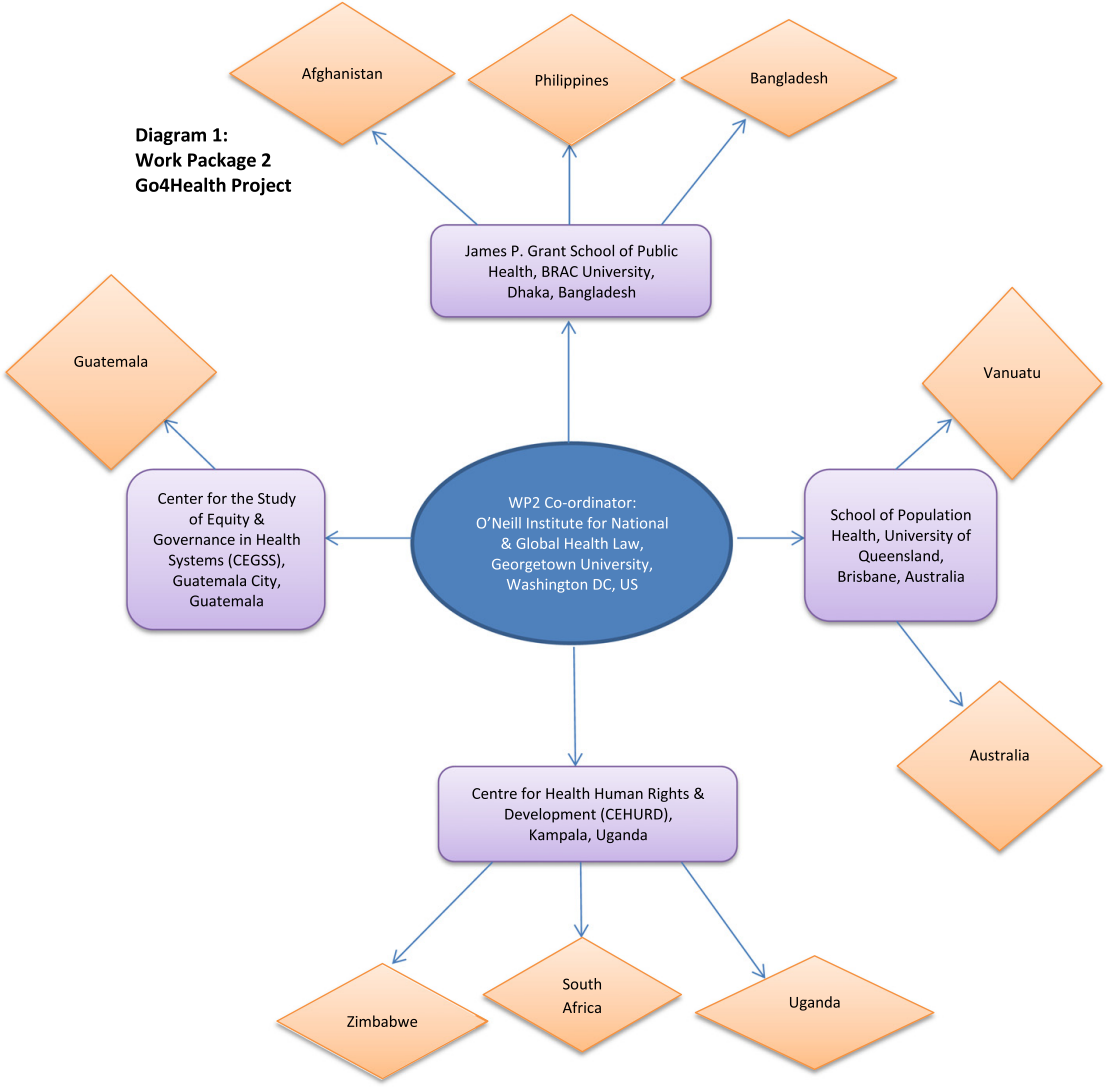
**Work package 2 Go4Health project.**

This article is a reflexive commentary on the methodological challenges experienced by Work Package 2 (WP2) of the Goals and Governance for Health research consortium (or ‘Go4Health Project’), which is undertaking community consultations around the world to inform recommendations on health in the post-2015 sustainable development goal agenda. WP2 is coordinated by the O’Neill Institute for National and Global Health Law at Georgetown University (United States) and research partners are located in both civil society and tertiary institutions in Bangladesh, Guatemala, Uganda and Australia (Figure 1). Initial work centered on a large-scale, qualitative research project with marginalized communities in nine countries in 2013.

The purpose of Go4Health’s widespread community consultations, mainly through focus group discussion and key informant interviews, is to ascertain a ‘snapshot’ of the health needs and priorities of socially excluded populations in low-income to high-income countries. This is to inform and ground Go4Health’s advice to the European Commission on the post-2015 global goals for health and new governance frameworks. Go4Health is “committed to ensuring that any post-2015 health and development goals are articulated in collaboration with the communities whose health is at stake” [1]. The norm of community inclusion and participation is well-recognized in the global health sector [2-4], and has been especially progressed by the “Greater Involvement of People Living with HIV and AIDS (GIPA)” principles of the AIDS movement [5] and People’s Health Movement [6]. Lay participation is fundamental for effective and sustainable public health interventions [7] and redressing paternalist practices accompanying ‘top-down’ development approaches [8]. It is also an integral element of the right to health.

### Community participation in formulating the post-2015 development goals

As the Millennium Development Goal (MDG) deadline materializes, negotiations are in full swing on the second iteration of global development goals. This “hurricane” [9] of post-2015 activity was unleashed by the United Nations (UN) in mid-2012, when it initiated efforts to facilitate eleven global thematic consultations and fifty (a number that would be greatly exceeded) national level post-2015 dialogues, as well as support widespread post-2015 community engagement [10,11]. The UN Secretary General also appointed a High-Level Panel of Eminent Persons on the Post-2015 Development Agenda (HLP), which in May 2013 suggested 12 illustrative goals; a foundation for ongoing post-2015 discussion [12]. This is in stark contrast to the 2001 MDG process, where goals were formulated by staff from a small UN interagency team [13,14] without consultation of communities or governments (notably from the Global South), and presented to the world in a UN report shortly thereafter [15].

Not only were the MDGs formulated from the top-down, but they contained a Western bias resulting in their occidental application to ‘the other’ in ‘those’ lower income nations. Conversely, the HLP envisages the post-2015 goals to be “widely applicable” to all countries, irrespective of income status, thereby elevating the equitable development paradigm [16] promoted by the 2011 World Conference on Social Determinants of Health [17]. While continuing to redress extreme poverty and its antecedents, the post-2015 agenda also seeks to redress inequities experienced by the bulk of the world’s poor now living in middle-income nations, and the unconscionable disparities experienced by millions in high-income settings. The HLP also emphasised each goal must “Be grounded in the **voice of people**, and the priorities identified during consultations, especially children, youth, women and marginalised and excluded groups” [12]. Certainly, people *want* to participate, in both the post-2015 agenda setting *and* goal monitoring phase [18,19].

Go4Health’s consultative aspirations are far more modest than the UN’s aim to “gather the priorities of people from every corner of the world” [11]. Nonetheless, our empirical gathering of lay perspectives from the world’s marginalized are important because these voices are distinctly missing while others speak loudly in their absence, especially within the many contributions to the UN Global Thematic Consultation on Health. For example, of the 100 papers submitted to the Global Thematic Consultation on Health, the bulk of papers are from multilaterals, development and health organizations and networks, religious organizations, civil society organizations as well as academics [20]. While the content of these submissions are significant and cover a broad array of issues – they nonetheless reflect organizational positioning, interests and priority areas, while the voices of the people most likely to be affected by the post-2015 development goals are absent. For a number of reasons it is not surprising lay perspectives are absent from the submissions: Community engagement to elucidate such voices takes time (far longer than given in the Global Thematic Consultation’s call for papers) and involves substantial human and fiscal resource; individuals may be unable to contribute to the submission process for technological as well as lack of English language and literacy reasons; and, of course, many communities lack awareness of the post-2015 health and development goal agenda (and its importance).

Further, although some 88 country consultations on the post-2015 agenda have taken place (or are occurring), question remains around timeframes as well as breadth and depth of the UN’s community consultative process. Indeed, it is unclear how or if the inherent richness in that data will be further analyzed, synthesized and brought to global attention (especially the attention of the intergovernmental Open Working Group on Sustainable Development Goals, a key post-2015 negotiator [21]). Nevertheless, the UN’s emphasis on equity and community participation in formulating the post-2015 development goals is a laudable shift from the pre-MDG era.

### The reflexive process

As qualitative researchers involved in this complex international project, WP2 investigators recognize they are integral to the research process and their scientific approach must be reflexive [22]. This means WP2 researchers “constantly take stock of their actions and their role in the research process, and subject these to the same critical scrutiny as the rest of their ‘data’” [23]. When researchers locate themselves within their research they embed greater accountability into their scientific practices [24]. In view of the diversity of investigators, and in order to identify, analyse, and learn from the ethical challenges experienced by WP2 in its early research planning and execution phases, WP2 implemented a “reflexive action learning” approach [25]. According to Richman et al. [25], action learning is “uniquely responsive to research environment and context” and this “evidence-based approach to continuous learning and reflection” is ideal to apply when collaborating with a group of colleagues. In this spirit, a WP2 team member (CEB) reviewed group meeting notes from two face to face preparatory meetings, Skype meeting minutes and project discussion notes from WP2 WebEx (an Internet-based communications platform) meetings, and re-examined group email correspondence over a 6 month period (September 2012 – May 2013) between WP2 partners. CEB then undertook the first round of data analysis and developed an initial paper, which the WP2 team commented on through email correspondence. The paper then served as a base for focusing critical discussion on research approaches at the third preparatory meeting in Heidelberg, Germany, in May 2013. Following this meeting, WP2 members revised the original draft, which was again circulated to the team for critical feedback. Subsequently, five main methodological challenges were identified, and are discussed below.

### Five main methodological challenges

#### Meanings and parameters around qualitative participatory research

An ethical approach to accessing marginalized populations brings its own complexities. WP2 researchers are entering into a relationship with populations for whom the focus of the research has no immediate local benefit, and for whom it may not have immediate relevance. Indeed, certain groups were reticent to engage due to prior interface with other researchers without long-term community benefit. This included potential participants from indigenous communities in Australia and Guatemala. Various contextually responsive strategies were thus identified: Investing additional time in building relationships with communities prior to research commencing [26]; partnering with appropriate community-based research workers and civil society organizations who are already existing members of the focal community; locating additional resources to integrate Community Based Participatory Research (CBPR) approaches [27]; connecting the consultations process to already ongoing community and national processes; and returning to communities to discuss findings and identify issues of (and partners/resources for) potential advocacy.

Table 1 provides seven “desired features” sought to be achieved within WP2’s research approach, and WP2’s ensuing efforts (and limitations). This Table describes how much engagement was desired and possible, versus what was achieved.

**Table 1:** Community engagement with marginalized populations: Desired features* and WP2 efforts

**Desired features**	**WP2 consultations**
Diversity of communities	•Within budget, time, and other imposed constraints, we were able to consult a diverse set of communities, encompassing indigenous populations, rural poor, ethnic minorities, people with disabilities, LGBT individuals, refugees, migrants, older adults, and youth (among others) in different geographic regions of the world.
	•Consultations with urban populations, outside of Africa, were limited, and more were planned. For example, a planned consultation with slum dwellers in Dhaka, Bangladesh, was precluded due to political unrest at the time.
	•Several consultations included a particular focus on women, especially in Asia and the Pacific.
Inclusion of highly marginalized populations	•All hubs sought to include highly marginalized populations in the consultations, including the development of a protocol for this very purpose in Guatemala, as well as for several of the highly marginalized groups in Uganda, namely LGBT individuals, people with disabilities, older persons, and post-conflict communities. For the most part we believe we succeeded, though in several of the communities, particularly where consultations were not linked to ongoing processes, we can be less certain of this (notably the Australian context).
Community participation at early stage of consultations	•The specific mandate of the Go4Health project addressed terms set by the European Commission as funders, limiting the potential to engage community members in the design of the project or the research tools. Community views on the post-2015 health development goal(s) were central to WP2. WP2 is committed to ensuring community voices and perspectives are heard, and remain responsive to community requests regarding feedback and ongoing representation and interaction.
Opportunity for all participants to have their say	•We strove in all our consultations to ensure that everyone could have a say. This included holding separate focus groups for LGBT individuals in Uganda, as discrimination made it unlikely that participants could speak out in a more open forum.
	•To foster participation in Asia, we held separate consultative sessions with specific populations within the community, in particular males and females from different age groups, including older persons, adults of reproductive age, and adolescents.
Findings shared and checked with community	•We shared findings with communities where funding enabled us to return to communities once each hub had analyzed the findings. This was possible in about half of the communities, in particular, most of those in Bangladesh, Guatemala, Uganda, Zimbabwe, and the Philippines.
Link to ongoing processes and advocacy	•Links to ongoing processes varied significantly by region, largely determined by the extent of already existing relationships with the communities. These were deepest in Guatemala, Uganda, and Zimbabwe.
	•While less connected to advocacy, the connections between BRAC University and the BRAC NGO will enable our Asian hub to discuss findings with relevant members of the BRAC NGO. This will enable the consultations in Asia, some of which were with communities that received services from BRAC, to affect the services they receive from and their interactions with BRAC.
Link to national processes	•In several countries, particularly those in Africa, as well as the Philippines, it has been possible to facilitate participation of communities we consulted into national dialogues on the post-2015 sustainable development agenda. For example, in Uganda national processes were also linked not only to the post-2015 process, but also to citizen participation in health through Health Unit Management Committees and annual community dialogues for health to inform the budgeting process.

*This list is not intended to be comprehensive.

#### Representation of marginalization in WP2

While it was acknowledged that four research clusters cannot capture the extraordinary diversity of marginal human experience, question nevertheless arose over which countries and in-country groups should be involved. Recognition was had, however, that the construction of the research proposal, time and resource constraints, and the geographic location of each of the research hubs (and their respective networks) dictated what is possible in terms of research parameters; frequently the case in community-based research projects [28,29]. Marginalization was framed by the four research hubs based on existing literature and field experience. The individual approach taken by each WP2 hub in order to select marginalized populations was considered flexible and appropriate in light of the geographically and contextually differing circumstances within (and facing) each research team. In Guatemala, for example, the identification of the communities to be consulted was the result of a participative process where civil society organizations, experts and people on the ground were interviewed and involved in active discussions. In addition, the team also took into consideration the country’s recent history, health and other social indicators and the feasibility of engaging with a community in a way that would be sustainable and beneficial for both the consultation and the community’s on-going process of achieving better access to higher quality health services. A similar approach was utilized by the Centre for Health Human Rights & Development (CEHURD) in Kampala, Uganda, where a regional meeting was held at the inception of the consultations with civil society partners from Uganda, Zimbabwe, South Africa, Kenya, Zambia and Malawi to guide the research process. Even after the regional meeting researchers from Uganda, Zimbabwe and South Africa had another national process to domesticate collaboratively devised research tools and methods of entry to the communities.

In contrast, the PROGRESS acronym, which stands for ‘Place of Residence, Religion, Occupation, Gender, Race/ethnicity, Education, Socioeconomic status, Social Networks and Capital’, was utilized by the Bangladeshi research team from BRAC University to frame marginalization [30]. As the PROGRESS acronym was particularly useful and applicable across countries in different phases of development, it was helpful in supporting the Australian research hub identify potential participants located in a high-income country context (permanent citizens of refugee background and indigenous Australians were subsequently identified and included). However, in terms of identifying and accessing participants in Vanuatu, the Australian research team was guided by its research partner, Vanuatu’s Ministry of Health, based on past experience of culturally and politically appropriate research engagement within that country.

#### Generalizability of research findings

Concern was had around how the entire data set could be rigorously synthesized into meaningful research findings; how could there be scientifically legitimate meaning comparing responses from groups identified as marginalized in an urban Australian environment to those from a rural and post-conflict environment in Uganda. It was agreed that the contextual and geographic variations and the factors underlying them were important given the emergent universal framing of the post-2015 goal agenda. Team members recognized “…every contradiction, every inconsistency, every diversity” should not be perceived “as an error or extraneous but as fodder for contextual analysis” [31]; that WP2 results may “simply reflect multiple realities, and, if an appreciation can be gained of the *reasons* behind the variations, this understanding may prove as useful to the reader as the results actually reported” [32].

#### Ethical research in project time frames

Qualitative community-based research is time-intensive and hurdles unexpectedly arise [33-35]. Delays were experienced by the WP2 team on multiple fronts: institutional ethical clearance processes, researcher health, and finalizing participant involvement. In the conflict zones of Afghanistan and Mindanao (Philippines), WP2’s Bangladeshi research team and their in-country partners planned fieldwork was delayed (or alternatives swiftly found) for security reasons. Bombings in Kabul, for example, delayed travel to the field, and in Mindanao authorities instructed local community research workers could not partner with, but had to replace, the foreign research team in the field. In Uganda, once in the field research team members sought extra letters of support from the WP2 project coordinator in Kampala as well as proof that the European Commission was actually funding the Go4Health Project. This could be linked to current government desire to control political activity in communities. Indeed, approval for the community consultations to take place in Uganda was obtained from the National Council of Science and Technology (NCST), but this approval at the national level needed to be supplemented and supported by further clearance from the representative of the Office of the President in each of the districts where consultations occurred.

## Informed consent

WP2 researchers took a contextualized approach to obtaining consent: Based on the literacy (and previous research experience) of the participant community, some sought written while others sought verbal consent (or both). Studies highlight that despite adherence to ethical guidelines and the researchers’ best intentions, there can still be a lack of informed consent among participants, particularly those in low-resource settings [36,37], and that meaningful consent is a challenging, iterative, and participatory process [38-40]. WP2 members discussed the need to consult community research workers, investigate and apply optional iterative consent techniques (mindful that a number of communities involved value a collectivist worldview), and seek to ensure that participation did not happen under any form of duress. In Vanuatu,for instance, a predominantly patriarchal society [41], the research team worked with local tribal groups and leaders to progress separate focus group discussions with female participants, confirming consent to participate with both the (male) leaders and, separately, with the individual women. In Guatemala, group discussions were favored over individual interviews to respond to the cultural views of the consulted communities. Talking to a group of community members allowed for the emergence of rich discussion while also ensuring support for the participants that had come to share their experiences of the neglect, discrimination and outright abuse that either they or their families had experienced.

## Conclusion

This reflexive analysis of WP2’s early research is unusual. Such analyses are typically published once the entire research project has been completed. However, the Go4Health Project is atypical: We are unaware of a comparable multi-country qualitative study undertaken by university and civil society partners on the post-2015 health and development goal agenda. As a multi-regional and multidisciplinary collective, undertaking research with logistic and methodological complexity, there is commitment to engage in reflexive analysis to identify successes and shortcomings of WP2’s practices at all research stages [42,43]. It is hoped that by reporting on these early challenges (and not waiting until the project finishes at the end of 2015, when the new post-2015 goals have been formulated), other similar projects, whether they involve partners at a more local level, may also come to light and share their post-2015 community engagement experiences. We advocate for a discourse to emerge not only critically examining *how* and *whose* voices are being obtained at the community-level to inform the post-2015 health and development goal agenda, but also how these voices are being translated and integrated into post-2015 decision-making at national and global levels.

## Abbreviations

CBPR: Community based participatory research
GIPA: Greater Involvement of People Living With Hiv And Aids
HLP: High-Level Panel of Eminent Persons on the Post-2015 Development Agenda
MDGs: Millennium Development Goals
UN: United Nations
WP2: Work package 2
